# A Sketch of the Origin and Present State of Medicine, and of Medical Institutions in Russia

**Published:** 1836-04

**Authors:** George Lefevre

**Affiliations:** late Physician to the British Embassy at St. Petersburg, &c.


### PART FOURTH.

### Medical Intelligence.

---

### SKETCH OF THE ORIGIN AND PRESENT STATE OF MEDICINE, AND OF MEDICAL INSTITUTIONS, IN RUSSIA;

By George Lefevre, M.D., late Physician to the British Embassy at St. Petersburg, &c.

### PART I. INTRODUCTORY.

It must be interesting to our countrymen to find that, in the remotest periods to which the origin of medical institutions in Russia can be traced, the foundations were laid by the English faculty; and that the sacred persons of the Czars were intrusted to the care of British physicians, when the art of medicine, as regarded the country in general, was exercised by the clergy alone.

How little influence soever the British may now possess, and how much soever even that little may be upon the decline, still Britain may boast that one of her countrymen is attached to the imperial person, that an English accoucheur has presided over the births of all the imperial children; and, last not least, that the medical institutions of Russia have been formed, and are presided over, by a British subject. It is to the latter individual, viz. Sir James Wylie, that Russia is indebted for the organization of her medical schools, both civil and military; and it has been by his persevering industry that the Medical Academy of St. Petersburg has arrived at the honorable rank which it now holds among medical institutions. Military medicine, in particular, is most indebted to his services; and it is not necessary to refer to very remote periods to shew, by comparison, how much he has done for the wounded soldier.

In the year 1600, there was only one surgeon attached to each regiment containing 1600 men; and the cost of a cavalry regiment for drugs, in 1676, amounted only to forty silver rubles, or about 9*l.* sterling, annually. Even in the present century, at the close of the last war, there was no medical staff; and an officer of rank, wounded on the field of battle, was allowed to take away with him the only efficient medical assistant; but, in the latter campaigns, an abundant supply of medical officers was furnished to the military and naval forces. The common soldier has to thank Sir James Wylie for such care and protection as his predecessor in arms demanded in vain, and the army in general has to thank him for a real and effective, instead of an inefficient and nominal, medical staff.

All institutions in Russia, which are under the protection of the crown, bear more or less the military stamp; and the Academy of Medicine, although a civil institution, is presided over by Sir James Wylie, who unites in his own official capacities the two supreme medical posts in the empire. The difference, therefore, between civil and military institutions is more nominal than real, and, as far as regards the education of students and the school of medicine, there is no practical difference.

Until a recent period, the native Russian was not only destitute of medical advice, but was almost independent of it, contenting himself with such artificial means as chance had discovered to him for the maintenance or restoration of health. Among the chief of these was, from the earliest times, the use of the vapour-bath, which, employed by the people even from their tender infancy, was one of the most essential means of strengthening the body. They were not, however, unacquainted with the virtues and uses of drugs and other remedies. It is stated, and upon good authority, that the use of sublimate dissolved in brandy was known at a very early period in Russia, even before Van Swieten had made it known to the rest of Europe. Their vapour-bath also was not merely employed for purposes of cleanliness and as a preservative of health, but used in cases of sickness as a remedy. The external applications employed in those days were of the most active kind. The actual and potential cautery and dry cupping were in vogue for the cure of local affections, and the highest personages of the state submitted to the application of these rude remedies. Still there is no mention made of a medical faculty authorising and directing such operations, or of any physician or surgeon of eminence.

The first physician, or at least the person who lays claim to that title, was Johannes Swera, a Pole by birth, who was attached to the person of Vladimir the First, and sent by him into Egypt, where he was baptised a Christian in 990. Of his medical abilities nothing is known; he seems to have devoted his life to religion.

The progress of medical science was arrested for many successive years after this period by the wars carried on against the Tartars; and the horrid plagues and famines, which for so long a period desolated the empire, prevented the influx of scientific men of all classes from abroad.

In the year 1128, the first traces of a medical police are to be found in Russia: this comprised little more than the appointment of certain persons to carry the dead bodies out of the town of Novogorod, and bury them in the environs.

The unhappy fate of Dr. Leo, a Venetian Jew, who accompanied Andrew, the brother of the Grand Duchess Sophia, from Venice to Moscow, forms a memorable item in the medical history of this period. Soon after his arrival, says the historian Richter, Dr. Leo had an opportunity of proving his skill. Ivan Ivanovitch, son of the reigning prince, suffered from a species of gout in his foot. The newly-arrived doctor, presuming too much perhaps upon his own skill, promised the father of the young prince that he would cure the disease, and engaged, if he failed, to forfeit his life. He administered some simples internally, and applied locally glasses filled with hot water: unfortunately these measures had not the desired effect, and the patient died in his three and thirtieth year. As people in those days believed in the infallibility of medicine, so they ascribed the failure to evil disposition of the doctor, and he was publicly executed.

No mention is made of any foreign medical arrival for a considerable period afterwards. In the mean time plague and famine devastated the empire, and several diseases, hitherto unknown, increased the wretchedness of those times: among these are enumerated the leprosy, the hooping cough, the English sweating sickness, the scurvy, the plica polonica, and the venereal disease. Of the dreadful ravages of the plague, the following account of it in the small town of Dorpat will furnish an adequate idea: "Insuper et pestilentia tam atrociter sæviebat ut anno 1551, Torpati trimestri spatio 14 millia hominum morerentur. Tanta erat ægrotantium multitudo, ut in cæmeteriis, in campis, in propatulo passim miserabiliter decumberent."*

Forgetful of the fate of Dr. Leo, or zealous in the desire of combating these new diseases, foreign physicians began again to creep into the empire. Two Germans, of the names of Theophilus and Lujeff, were called to court in 1534, and desired to treat the Prince Vassilig Ivanovitch for a carbuncle; and boldly attempted to cure the disease, but were unsuccessful. The prince, finding no amendment, sent for Dr. Lujeff, and asked him if it were possible to cure him? to which question the doctor made this reply: "My lord and master,—when I was at home in my own country, and heard of your great goodness and liberality, so quitted I my home, even my father and my mother, to come and see

* Breodenbach Historia Belli Levanici in Rev. Moscow.

you; but can I raise the dead, as I am not God Almighty?" Upon which the prince turned to his courtiers, and said, "Nicholas has passed sentence upon me!" The fate of the Prince in this instance brought no discredit upon the doctors.

There was still a demand for foreign talent, and the Czar wrote to Charles the V., and to the Emperor Rodolph the II., to beg them to send artists and learned men of all descriptions to Russia.

Now commences also the æra of the influence of the British faculty in Russia; an influence which preponderated greatly over that of other nations for the space of two centuries.

The celebrated navigator, Sebastian Cabot, fitted out an expedition for the discovery of a North-West Passage to China and India. Three of the ships set sail from England on the 20th May, 1553, under the command of Hugh Willoughby. A furious storm caused them to part company: one of them went to the bottom. Willoughby reached the harbour of Arzina, in Russian Lapland, and was frozen to death, with all his crew. The *Bonaventura*, commanded by Richard Chanceller, arrived in the White Sea, and got into the bay of St. Nicholas, which formed subsequently the port of Archangel. Chanceller was received at Moscow with much cordiality by the Czar, and returned twice to Russia, in 1555 and 1556. He was drowned on his return to England, in the last-mentioned year. This discovery of Russia, as it may be styled, was the means of opening a commercial intercourse between the two Countries.

Anthony Jenkinson, who followed Chanceller in 1557, brought with him a doctor; and during the reign of Elizabeth, this same Jenkinson *imported* at different periods no less than fourteen doctors and one apothecary.

Queen Elizabeth sent Dr. Jacob to Russia, and recommended him as being well skilled in female complaints, having often benefited herself by his advice, and she assured her beloved sister the Czarina, that he knew more about the situations of lying-in women than even the midwives themselves. Dr. Jacob was one of the physicians whom Anthony Jenkinson took to Archangel, and he was followed a few years afterwards by Dr. Marcus Rydley, who was chosen for the service of the Czar by Lord Burleigh, and was recommended as a man learned and expert in his profession. He was a Graduate of Cambridge and a fellow of the Royal College of Physicians. He was a great favorite at court, and remained four years in Russia. After the death of the Czar, he was recalled by Queen Elizabeth, and permission was granted him to return to his native country by the Czar's successor; who, at the time of taking leave of him, gave it to be understood that, if in future any English physician, apothecary, or other learned personage, should desire to come to Russia, he might depend upon a kind reception, a due maintenance, and free permission to return home.

Relying upon these promises, Dr. Willis accepted the conditions in 1593. Hardly had he arrived, however, before he was very ungraciously sent out of the country, being accused of meddling with politics; and the Queen herself received an injunction to forbid her ministers sending any such man in future to the court of Moscow. It is but justice to his memory to assert, that his medical talents were not called in question; but a singular charge was brought against him, that he did not bring with him a sufficient stock of books. The Queen took up his defence very warmly, in a letter which she herself wrote to the Czar; and, touching his meager supply of books, she observes, that the doctor, in reply to this charge, answered, "that hee sent both his bookes and drugs along by sea, and hee himselfe travayled over land, by which passage he could not have convoyance for those things, &c. &c."

Of John Fensham, the apothecary, it is stated that he brought with him a great quantity of drugs; and, in the list of those he imported, the Confectio Eryngæ figures at the top; the people of Eastern origin formerly ascribing particular virtues to this remedy.

The oldest medical manuscript in the Russian language bears the date of this period, 1588. It is a translation from the Polish, contains 1561 folio leaves, and savours not a little of the ignorance of those ages. The influence of precious stones had much credit in those days, and the author observes, that garnets rejoice the heart, that the magnet grows in the great Indian Ocean, and he who carries about with him rubies will have no frightful dreams.

The influx of foreign physicians from all parts of Europe into Russia, and the necessary admission of some adventurers, furnished with false diplomas, and totally disqualified to practise their profession, arrested the attention of the legislature, and in the reign of the Czar Michael Feodorovitch (1613-1645), a Board of Examination was instituted for proving the qualification of strangers who came to practise the medical profession in Russia. Under the titles of Apothecary's Hall, Apothecary's Chancery, Medical College, Physicant, &c., it has with some trivial changes been transmitted down to the present time. It was originally composed of doctors belonging to the court, and of some of the high dignitaries of the state; and all physicians and apothecaries without exception were subjected to its control.

The principal duties consisted in ascertaining that the imperial pharmacy was well supplied with drugs; in appointing army surgeons, and supplying military medicine chests; in paying the yearly salary of the medical officers, and in settling all disputed claims. All newly arrived physicians were ordered to announce their arrival at the foreign office, from whence they were directed to this tribunal, where they underwent an examination to prove their qualifications, and receive their different appointments in the Czar's service. It was the privilege of this office to appoint medical men to attend upon the sick nobility and wounded military, and there was no appeal against its decisions.

It was also its especial duty to watch over the health of the inhabitants and prevent the propagation of contagious diseases. Still, however, no quarantine laws were established during the reign of Michael Feodorovitch; and, what is still more striking, is the circumstance that, up to this period, and for a long while afterwards, Russia was never so free from plague and pestilence as during the long reign of this Czar. It was considered sufficient in those days, when a traveller arrived at the gates of the city, to enquire of him, if he had encountered the plague in his travels, and to make him indicate the place where he had observed it. Under this Czar an ukass was published, (in 1637,) forbidding the execution of pregnant women; and respiting them till six weeks after the birth of their offspring. Another order was issued for devising the best means of arresting the murrain among cattle; and this was achieved by the vigilance of a well-directed medical council.

It was during the reign of this Czar, also, that our countryman, Dr. Arthur Dee was invited to reside at the court of the Czar's; and he accompanied the Russian Ambassador to Moscow in his medical capacity. We find no less than four royal epistles written on his account; and no physician in those or even in later times was ever so well paid or received such presents. Independently of his salary and other perquisites appertaining to his situation at court, he enjoyed the revenues of an estate near Moscow for many years, and he possessed a brick house in that city sixty fathoms long and forty fathoms wide, which he was afterwards permitted to sell to his countryman, Simon Digby. Upon his return to England, he was appointed body physician to Charles I.; and, after the death of that unhappy monarch, he quitted public practice, and devoted the rest of his days to the discovery of the philosopher's stone. It is no where stated, says Dr. Richter, that he furthered the progress of medicine during his long sojourn in Russia, although he published a work in Moscow, with the title "Fasciculus chimicus, obstrusæ Hemeticæ scientiæ ingressum, progressum, coronidem explicans."

The name of Dr. Dee was afterwards quoted by his countryman Dr. Anthony to sanction the employment of some chemical preparations, which he sent out to the Czar, "highlie cordiall of excellent vertue, and singular use for prevention and for cure of most infirmities." These were a tincture of gold and a preparation of gold in powder,

His successor, Dr. Bremberg, a Dutchman, came to Russia uninvited, professing to cure most diseases by secret remedies, and despising the faculty individually and collectively. He produced testimonials of having performed wonderful cures; as, 1. That he had saved the hand of an Englishman, and healed it by simple means, after it had been condemned to be amputated by other doctors. 2. That he had taken off a middle finger without using the saw. 3. That he had been instructed by the best masters how to cure ruptures. 4. That he knew the secret of dissolving the stone in the bladder by internal remedies, and of expelling it afterwards. 5. That he first observed how useless the operation of trephining was in wounds of the skull, and had discovered a much less cruel method of cure. 6. That he could cure the gout in a superior style, and all other human infirmities. All these advantages, however, availed him nothing: he was sent out of the country, and branded as a quack.

Passing by several artists of minor note, we find that the reputation of our countryman, Dr. Peter Chamberlayne, had attracted the attention of the Czar, who wrote with his own hand a letter to Charles I., begging him to allow the doctor to enter into his service, understanding that he was willing to do so. Great preparations were made for his reception at Archangel, which was the way from London to Moscow in those days; but a letter arrived from the king, excusing himself for refusing the Czar's request, upon the grounds that, as Dr. Elmston, a native Russian, had been studying medicine in England, and had returned to his own country, so was he capable of filling the office of body-physician to the Czar.

Previous to this period, the Russian government tried the experiment of supplying itself from its own resources; exporting native Russians to study abroad, and qualify themselves for exercising the healing art in their own country. To fulfil this intention, three young men were sent to study medicine in Holland and in England, and were supplied very liberally with funds for this purpose. Elmston was one of these, and he remained thirteen years in England, prosecuting his medical studies; but neither of him nor of his two colleagues is any honorable mention made after their return to their own country.

The ravages of the plague, which raged in Moscow in 1656, led to the establishment of rigid quarantine laws. All communication was cut off with the city, and a violation of the law was punished with death. No persons from the neighbouring towns were allowed to go there. All travellers proceeding thence were detained, examined, placed under the observation of the police, and only allowed to speak with the inhabitants at prescribed distances. The clothes of the sick were ordered to be burnt, and those which were not destroyed were fumigated. The doors of the cottages were left open, and the chambers thus exposed for a fortnight to the severity of the cold, after which they were fumigated with wormwood during three successive days. All the official papers that arrived from Smolensk, which still suffered from the plague after it was stayed in Moscow, were copied afresh, and the original manuscripts burnt.

When the plague broke out in London only a few years afterwards, so great was the fear in Moscow of its being again imported into the city, that Dr. Wilson, who arrived from England the same year, was obliged to undergo several ablutions, and not allowed to go into the capital till some months after his arrival.

The mystical revelations in those days, and the universal belief in astrology, and the influence of the stars in earthy affairs, had great weight with the government. By command of the Czar, many questions were put to the physician and astrologer, Dr. Engelhart, who was in Moscow at the time, concerning the impending variations of the ensuing year. Upon which he predicted in his reply, in December, 1664, that a dreadful plague would sweep over other countries besides Russia. The verification of the prophecy, as regarded England, added to his reputation, and the government redoubled the rigidity of all the quarantine regulations. The port of Archangel was closed, and all communication cut off with England.

Of all the physicians hitherto known in Russia, Dr. Samuel Collins is reputed to have been, without exception, the most celebrated for his writings. He was a graduate of Oxford, and accompanied the imperial Commissary Gebdon to Moscow, who had been sent to Holland and other countries in order to procure celebrated men for the Czar's service. He was contemporary with Dr. Engelhart, but, unlike his colleague, he turned his attention to the demonstrative sciences, and wrote a work upon anatomy, which gained him the commendations of Haller. He practised eight years at the imperial court, and received great honours and rewards. He remarks that, during the fasts, physicians were not allowed to prescribe any substances from the animal kingdom; so strictly was it forbidden to take any thing animal into the stomach.

The feeble health of the Czar Feodor Alexevitch obliged him to have frequent recourse to his physicians. There was a distrust in his courtiers of the loyalty of the foreigners about his person; and, as all the doctors and apothecaries were strangers, fears were entertained that they might be instigated to administer poison to the Czar or to some of his family. Dr. Rosenberg, the chief physician at court in that reign, stated that he himself saw an apothecary forced to swallow the remainder of a potion, in the preparation of which he was supposed to have committed some error. It was the custom in those days in Russia, as it is in Persia at present, to make some of the persons about court swallow a portion of every medicine prescribed for the sovereign. A lady, who had taken some physic intended for the Czarina was suddenly taken ill, and suspicions fell upon the apothecary. He was not only obliged to swallow his own medicine, but was sent into exile, although he protested his innocence, and declared that both he and the physician who prescribed had always taken a portion of every medicine which was destined for the Czar or for any of the imperial family.

An oath was now administered to the different persons about the Czar, and to the doctors in particular, by which they swore not to put poisonous herbs or roots into the victuals or clothing of the imperial person. It was enjoined, that every recipe should be translated into Russ, and deposited in the chancery; and the name of the doctor and the patient, with the date of the day and month, were all registered in a book.

The next physician of note was sent to Russia by the Emperor Leopold, at the request of Peter the First. He was a Greek by birth, and was the first who introduced the inoculation of the small-pox into Europe. He published a work under the title "Nova et tuta variolas excitandi per transplantationem methodus." He seems to have divided this honour with Emanuel Timon, who flourished about the same period, and wrote a work with the title "Historia variolarum quæ per incisionem excitantur."

The experiment of sending native Russians to study medicine in foreign countries was again tried by Peter I., who sent Posnikoff, the son of a Russian nobleman, once Ambassador in England, to attend the schools of Italy. He graduated with great honour at Padua in 1696, and took degrees in philosophy and medicine. He was subsequently attached in his medical capacity to several Embassies, and returned to Russia, in 1701, to practise his profession. He received a salary of five hundred silver rubles from the court; but, of his future medical career, nothing is handed down.

The regeneration of the Russian empire under Peter the Great was the dawn also of the organization of medical as well as of scientific institutions, and the Academy of Sciences, which has by degrees raised itself to some eminence in Europe, was founded by that monarch, and completed by his widow. The first president of the Academy was Dr. Blumentrost, the body physician of the emperor.

No mention is made in Russian history of institutions for the sick and infirm, till about the year 1682, when a private gentleman, of the name of Rtischeff. bought a house, and converted it into an hospital for the reception of from thirteen to fifteen invalids. Rtischeff was the Howard of Russia, and devoted his time to visiting the prisoners, ameliorating their condition, and releasing many by paying their debts. His example was soon followed by the government, and the reigning monarch issued an order for the establishment of a large hospital in Moscow. The endowment of this hospital led to the establishment of a school of surgery: it was placed under the superintendence of a governor: a physician, three or four surgeons, and several students, attended the wards. In the course of time a pharmacy was attached to it. The funds were supplied by the revenues arising from the lands of the former archbishop of Archangel, and from the money deposited in all the churches, in the poor's box. The owner of the boor was then, as now, obliged to pay for his maintenance whilst in the hospital; but in those cheap times any individual might rent a bed, or any number of beds, at the moderate price of ten silver rubles per annum, which bed he could always keep occupied. This practice still exists, with the difference only that each boor pays at the rate of four silver rubles per month, whereas the free man is admitted gratis. This seeming anomaly is explained by the circumstance that the owner of the slave is obliged to provide for him in sickness, and consequently, if there were no such regulation, the hospitals would be crowded with all the sick and infirm boors, and the poor freemen would be excluded from such advantages.

Peter the Great soon commenced preparing a receptacle for his wounded seamen, after the famous battle of Narva. In 1706, a military hospital was erected in Moscow. It is supposed that the idea was suggested to him when he visited Greenwich, in 1698. He established within its walls an anatomical theatre and a school of surgery.

The monarch was about to create a new city in his empire, and one of the first public buildings was a naval and military hospital, founded in the year 1715, of which the foundation-stone was laid by the monarch himself. The hospital was opened for the reception of wounded soldiers and sailors in the year 1720, and it contained as many as 500 invalids. To supply funds for its maintenance, it was ordered that, upon every military promotion, each officer, whatever his rank, should deposit a month's pay, annually, into the hospital fund. Within the walls of this same building was originally established, and still exists, the Medico-Chirurgical Academy and the School of Medicine of St. Petersburg.

Peter not only studied, but actually practised, the healing art. In the year 1698, he studied anatomy in Leyden, and subsequently in Amsterdam, under the celebrated Ruysch. "Gloriosum id manebit semper Ruyschium corporis humani fabricam exposuisse Petro Primo Magno Russorum Imperatori, qui intentissimo auscultebat animo, super omnibus rogitabat sedulo, atque habebat in memoria semper quod viderat semel." He attended the lectures on anatomy given by Bidloo in Moscow, and was often present at the dissections; and it is reported of his zeal, that he ordered the dissection of a half-witted page, who had died drunk, to be postponed till he could attend in person. His attention was particularly directed towards monstrous formations, and he ordered all that could be collected in the empire to be brought to St. Petersburg: hence the immense number which are still to be seen in the museum. He did not confine his studies to the theories alone, but was himself a practical surgeon, and always carried about his person two pocket cases, the one containing mathematical, the other surgical instruments. The latter was furnished with lancets, tooth-forceps, a saw-knife, a spatula, a pair of scissors, a sound and catheter, &c. He gave orders that he was previously to be informed of any remarkable operation, either in public or private; and, if it were possible, he attended and assisted in the performance. All the minor operations, as tooth-drawing, bleeding, bandaging, &c. he continually performed himself. A merchant who had an abscess in the foot was cured by the Emperor, who himself made an incision, and the operation was successful; but the circumstance coming to the knowledge of the Duchess of Mecklenberg, who had a similar disease, she left the city in post-haste, for fear of his assistance. The *chef d'œuvre* of his operations was the paracentesis abdominis, which he performed upon a merchant's wife, who had resisted the advice of her doctors, and would not submit to the operation. This coming to the ears of the emperor, he visited her, and persuaded her to let him perform the operation himself. In the presence of the faculty he drew off twenty-four pounds of water; but the operation was performed too late, and the patient died.

Under such auspices the medical institutions of course flourished. The Academy of Sciences was enriched by a collection of anatomical preparations, purchased for the sum of 30,000 guilders, and which had belonged to the celebrated Ruysch. During the reign of this great monarch, the foundations of hospitals, anatomical theatres, museums, and cabinets of natural history, were laid on a firm and solid basis. The giant and the dwarf of Peter the Great figure in this page of medical history, and their stuffed skins are still to be seen in the museum; for they were flayed after their death, and their skins stuffed like those of animals. It does not appear that this monarch intrusted his health to the care of any English physician; his favorite medical adviser was Bidloo, by whom he was attended in his last illness.

Although no British physician was immediately attached to the person of the emperor, if we except the surgeon Horn, who used to introduce the catheter, yet the greatest honours were conferred upon Dr. Areskin, (Erskine?) who, in 1716, was named Archiater of the Russian empire, and president of the whole medical faculty. In the history of this individual we have a second instance of English physicians in Russia being accused of political intrigues. Dr. Areskin was charged with the crime of favouring the Jacobites at the Russian court, and of being in correspondence with the Earl of Marr. He fully justified himself against all such charges, but died soon afterwards at Olonetz.

The army medical staff particularly engaged the sovereign's attention, and he issued an ukass specifying the number of medical officers to be supplied for the whole army. Each division was now furnished with a physician and staff-surgeon. Each regiment had a surgeon, and every company a felcher (surgeon's mate). Two military medicine chests were allotted to the whole army, and to each of these was attached an apothecary, two assistants, and four apprentices. The same arrangement was introduced for the service of the fleet.*

We must not omit mentioning a lithotomist, who came to Moscow from Constantinople during this reign. The Czar Michael Feodorovitch suffered from a stone in his bladder; and he requested the Sultan Ibrahim to send him an artist from Constantinople. It is not recorded that this request was granted; but in the year 1716, a Greek, named Fotii Nicotajeff, who had practised as a lithotomist and hernia doctor in Bucharest, came to Moscow, and brought with him a pupil, Dmitri Minaja. They had both acquired the method of making the simple incision, after the manner of Celsus, without introducing the grooved sound as a guide. The son of this Dmitri Minaja still practises in Moscow as a lithotomist, and performs the operation empirically, but with considerable success.

During the succeeding reigns of Catherine the First, of the empress Anne, and of the empress Elizabeth, there is nothing particularly worthy of mention. The Academy of Sciences, founded by Peter the Great, was opened to the public by his widow, in 1726, and Dr. Blumentrost was appointed president.

During the reign of the empress Elizabeth, schools of midwifery were first instituted, and ten native Russians were sent to Holland to study medicine.

There is little mention made of British physicians, from the time of Peter the Great's death, to the accession of Catherine the Second. The tragical fate of Lestocq, the physician and favorite of the empress Elizabeth, may have had its influence in preventing medical men from seeking their fortunes in Russia. Accused of treason by those whom he had most served, Lestocq was tried, and

* Geschicte der Medecin in Russland, entworfen von Dr. Wilhelm Michael von Richter. Moscow, 1819.

condemned to death. His sentence was never put in execution, for the Empress would not allow it; he remained four years in prison, when his sentence was revised by the court, and he was banished to Siberia.* He was recalled by Peter the Third, and died at St. Petersburg, in 1767.†

Upon the accession of Catherine, British physicians again flourished at the court of St. Petersburg. The invitation sent to Dr. Dimsdale, to come to Russia and inoculate the Empress, was a marked honour conferred upon the British faculty.

In 1768 the small-pox was rife in Petersburg, and the Empress, with the courage which characterized all her actions, set the example of inoculation to her subjects, by being first inoculated herself. Matters were soon arranged for putting the proposal into execution, for the Empress was decided upon submitting to the experiment. Dr. Dimsdale requested the assistance of the court physicians; but the empress would by no means consent to any consultation, and gave her reasons as follows: "You are come well recommended to me; the conversation I have had with you on this subject has been very satisfactory, and my confidence in you is increased. I have not the least doubt of your abilities and knowledge in this practice; it is impossible that my physicians can have much skill in this operation: they want experience; their interposition may tend to embarrass you, without the least probability of giving any useful assistance. My life is my own; and I shall, with the utmost cheerfulness and confidence, rely on your care alone. With regard to my constitution, you could receive no information from them; I have had, thank God, so good a share of health, that their advice has never been required; and you shall, from myself, receive every information that can be necessary."‡

After the operation was performed, the Empress went to Tzarsko-seto. It was done privately, and without the knowledge of the court; but as several of the nobility followed her, and as she observed some whom she suspected not to have had the small-pox, the Empress said to Dr. Dimsdale, "I must rely on you to give me notice when it is possible for me to communicate the disease; for, though I could wish to keep my inoculation a secret, yet far be it from me to conceal it a moment, when it may become hazardous to others."

The Grand Duke, shortly afterwards, submitted to the operation; and on his recovery, Catherine rewarded the services of Dr. Dimsdale by creating him a baron of the Russian empire, and appointing him counsellor of state, and physician to her imperial majesty, with a pension of £500 a year to be paid him in England, besides £10,000 sterling, which he immediately received.

The examples of these illustrious personages had such immediate influence, that most of the nobility, both of St. Petersburg and Moscow, were impatient to have their families inoculated. On the 3d Dec. 1768, a thanksgiving service was performed in the chapel of the palace, on account of her majesty's recovery, and that of the Grand Duke, from the small-pox. The metropolitan delivered an impressive discourse, in which he celebrated the resolution and magnanimity of the Empress; and in the course of the sermon, remarked, "that the Russians had borrowed assistance from Britain, that island famed for wisdom, bravery, and virtue."

Before Baron Dimsdale left St. Petersburg, an inoculation hospital was established for poor children. This institution was superintended by Dr. Halliday, an English physician, who practised a long time in Russia. When the pestilential disease broke out in Moscow, and caused such sad disturbances, this gentleman was sent by the Empress to report upon the nature of the malady. He performed his task so much to the satisfaction of Government, that he was rewarded with a handsome pension for life.

This was not the only hospital founded by Catherine the Second; to that

* Tooke says to a village near Archangel—Coxe says Siberia.

† Tooke's reign of the empress Catherine the Second.

‡ Idem. Tooke.

sovereign is due the finest establishment of its kind in Europe, viz. the Foundling Hospital of Moscow. The plan of this institution differs from the foundling hospitals and enfans trouvés of other countries. It is a reception for illegitimate children, it is true; but it admits children born in wedlock, of parents who are too poor to rear their offspring: hence it is styled Maison Impériale d'Education. Nothing can equal the care and pains bestowed upon every department of this establishment.

Other hospitals and medical institutions, both in Moscow and St. Petersburg, were founded by the Empress, who, during her long and glorious reign, encouraged everything connected with the arts and sciences.

Her body physician was the late Dr. Rogerson, a great favorite at the court of St. Petersburg. He lived and practised amongst the nobility, and the success of his career speaks well for his merits. He retired from service with such a well-requited reputation as falls to the lot of few.

Beyond this period, which terminated with the death of Catherine, in 1796, I have not thought it advisable to proceed with the biographical part of the sketch that I have proposed furnishing. It has been the lot of the distinguished individual mentioned in the first pages to create a new æra in the annals of medicine in Russia; and it remains to speak of the medical institutions as they now exist, assigning to him the credit that they are as they are.

Mr. Tooke, in his work entitled "The Reign of Catherine," has given in his last chapter a summary account of the state of the arts and sciences at St. Petersburg during the reign of that sovereign, and has not omitted the art of medicine. Even medicine, says the author, was but lately in so uncultivated a state, that, in the year 1770, perhaps there were not three books on medical subjects in the Russian language. Among the physicians who have deserved well of their country in this art may be cited Ambodik, professor of midwifery, who has eminently contributed to the enriching of Russian medical literature. He is the author of a plain and practical Manual of the Art of Midwifery, a Physiology, a Materia Medica, and an Anatomical Physiological Dictionary in Russ, Latin, and French. An enlarged and reformed translation of Saucerotti's celebrated Examen, under the title of "a Brief Examination of inveterate Prejudices and Notions concerning Pregnant Women, Lying-in Women, and New-born Children," a book that has already gone through several editions, is also from his pen. He is likewise the translator of Schreiber's "Guide to the Knowledge and Cure of Outward and Inward Diseases," and of Home's "Principia Medicinæ." Tissot's writings, "Avis au Peuple," and of the disorders incident to the Learned, are also translated into the Russian, by Drs. Ozeretzkofsky and Schumlianski. Several small pieces by Tichorsky, Dr. Mead's Dissertation on the Plague, Van Swieten's Description of Camp Sickness, and Baron Dimsdale's Method of Inoculating the Smallpox, have all been translated by Russian physicians. Dr. Bacheracht, a German, published a popular book on several diseases, another on Intemperance in Sensual Enjoyments, a Proposal for Preserving the Health of Seamen, a Treatise on the Scurvy, &c. M. Vien, secretary of the College of Medicine, published a very complete Loimology. The Privy-counsellor Peken is the author of a Physiology and Pyrethology for use at lectures, and the translator of Richter's Elements of Surgery. Spedikati wrote a controversial piece on the Scurvy, against Bacheracht. A translation of the Institutions of Gaubius was published by Professor Hoffman.

*St. Petersburg, Dec.* 1835. G. L.

